# Catalyst Deactivation in Syngas Tar Cracking: A Multianalysis Study of Coke Deposition on γ‑Al_2_O_3_‑Supported Iron Catalysts

**DOI:** 10.1021/acsomega.6c01836

**Published:** 2026-06-16

**Authors:** Francesco Parrillo, Vincenzo Arconati, Carmine Boccia, Umberto Arena, Filomena Ardolino, Giovanna Ruoppolo, Ange Nzihou, María González Martínez

**Affiliations:** † University of Campania Luigi Vanvitelli, Department of Environmental, Biological, Pharmaceutical Sciences and Technologies, Via Vivaldi 43, Caserta 81100, Italy; ‡ Institute of Sciences and Technologies for Sustainable Energy and Mobility, 9327 National Research Council-CNR, P.le Tecchio 1, Naples 80125, Italy; § 52866 Université de Toulouse, IMT Mines Albi, RAPSODEE CNRS UMR 5302, Campus Jarlard, Albi Cedex 09 F.81013, France

## Abstract

Catalyst deactivation caused by coke deposition remains the main limitation to the catalytic removal of tars in hot syngas cleaning. This study investigates the characteristics of coke layers deposited over two iron-based, alumina-supported catalysts. The first, Fe/γ-Al_2_O_3_, just doped with a market-available iron active phase; the second, RM/γ-Al_2_O_3_, uses red mud obtained as a by-product of the Bayer process for aluminum extraction. Catalytic cracking tests were carried out at 800 °C, without and with steam, by using naphthalene as a model tar component. A multitechnique approach combining FT-IR, Raman spectroscopy, and SEM-EDX analyses was applied to fresh and spent catalysts to assess the role of coke formation and its features in governing the activity and stability of catalysts for tar reforming. FT-IR spectroscopy provided qualitative insights into the presence and evolution of surface functional groups. Raman spectroscopy was used to quantify the relative distribution of amorphous and graphitic coke under different operating conditions. SEM-EDX analyzed surface morphology and elemental composition, providing visual indications of carbonaceous deposits. The overall results show that Fe/γ-Al_2_O_3_ achieves the largest hydrogen yields due to its higher iron loading, even though, when steam was not used, it undergoes severe deactivation caused by amorphous and graphitic coke deposition. RM/γ-Al_2_O_3_ exhibits a lower hydrogen production but a slower deactivation, likely related to the high content of alkali and alkaline earth metals on its surface, which reduces coke deposits. The results provide useful information for designing more reliable catalysts for tar syngas reforming.

## Graphical abstract

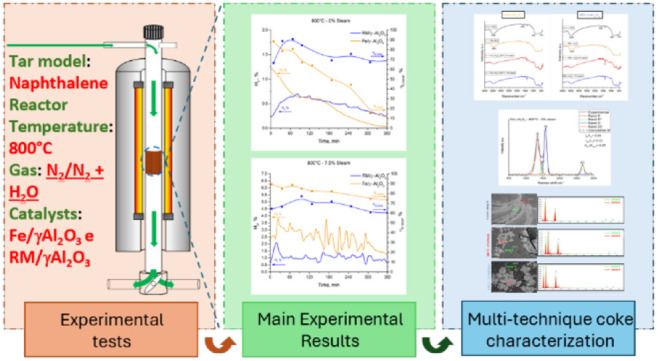

## 1 Introduction

The rapidly growing production of municipal solid waste (MSW), driven by urbanization, industrialization, and new consumption patterns, requires innovative and sustainable strategies for its management. In this context, gasification has received increasing attention due to its flexibility, lower environmental impact compared to other thermochemical processes, and its ability to treat nonrecyclable waste. The process converts carbonaceous solid waste into a fuel gas (syngas) through a series of heterogeneous and homogeneous reactions taking place in a reducing atmosphere. Syngas, based on its composition and the content of contaminants, can be used for a wide range of final applications, aiming at producing energy, fuels, and drop-in chemicals. It is composed of H_2_, CO, CO_2_, CH_4_, lower amounts of light hydrocarbons, particulates, and various organic and inorganic impurities. Among the different contaminants, tars—defined as hydrocarbons with a molecular weight higher than that of benzene—are the most challenging to deal with. Their high condensation temperature causes fouling, corrosion, and the formation of contaminated wastewater streams.^1^ Therefore, tars must be effectively removed to enable the utilization of syngas in high-value applications.^2^ Catalytic treatments consequently have attracted particular attention in recent years since they involve the decomposition of heavy, high-molecular-weight tar compounds into lighter and more stable gases, including H_2_ and CO, and allow recovering tar chemical energy. Moreover, they can operate at temperatures lower than those necessary for thermal cracking, so improving energy consumption. However, their use is associated with important technical and operating challenges. The most significant are those of catalyst deactivation related to some potential causes, like the sintering phenomenon at high temperatures (which involves the irreversible agglomeration of the catalytic particles), poisoning by contaminant compounds present in the syngas (such as sulfur), and deactivation caused by coke formation.^3^ To address these challenges, research is focusing on developing more deactivation-resistant catalysts that can withstand high temperatures and the presence of unwanted pollutants.^4–6^ Iron-based catalysts supported on stable oxides (e.g., Al_2_O_3_) are emerging as a promising option, as they can achieve high conversion and H_2_ yields while also being more resistant to sintering and poisoning deactivation.^7^ Furthermore, iron can be found in many industrial byproducts, making it an attractive option in terms of both economic and environmental sustainability.^2^ Nevertheless, coke deposition remains an unavoidable issue even for these systems, so effective mitigation and regeneration strategies are required to unlock their full potential. Coke is an unwanted byproduct of the tar cracking reactions: during the process, it builds up on the catalytic surface, hindering the contact between the reactants and the active elements and leading to a loss of specific surface area and activity. Coke-induced deactivation can occur within a few minutes, faster than the other, above-mentioned, types of catalyst deactivation.^8,9^ On the other hand, the process is reversible since coke can be converted using an oxidant such as O_2_ or steam, although it may not be removed completely.^10^ The chemical and morphological nature of coke, indeed, plays an important role, as it strongly affects its conversion kinetics. It can be amorphous—displaying an unordered crystalline structure, in which carbon atoms are arranged randomly, forming small, disordered, and unconnected microcrystalline regions—or graphitic—displaying carbon atoms arranged in a highly ordered structure of planar layers of hexagonal rings—as suggested by Parrillo et al.^2^

The coke morphology refers to how carbon is deposited on the catalyst surface. This can have different impacts on the process performance. Some authors have identified three types of coke morphology: encapsulating, filamentous, and pyrolytic coke, depending on the operating conditions and the catalyst used.^11,12^ Encapsulating coke is produced at low temperatures and low H_2_O concentrations, covering the active metal and leading to rapid deactivation. Filamentous coke has a more structured morphology, it is produced below the metallic active sites, with structures that are typical of carbon nanotubes or carbon nanofibers: its catalyst deactivation is slower but could weaken its structure, until its breakdown. Pyrolytic coke is formed at temperatures above 600 °C, not necessarily only on the active sites but generally indistinctly covering all the catalyst surface, forming a layer that fills all the pores: it is more graphitic, then requiring higher temperatures to be converted by gasification or combustion.

The aim of this study is to elucidate how coke formation and its structural and morphological characteristics affect the activity and stability of iron-based catalysts during naphthalene decomposition in high-temperature gas streams. Two different Fe-supported catalysts were used, under both dry and steam-assisted conditions, to assess the influence of steam on coke formation and catalyst stability. Raman spectroscopy, Fourier transform infrared spectroscopy (FT-IR), and scanning electron microscopy combined with energy dispersive X-ray analysis (SEM-EDX) provided a comprehensive characterization of the coke species formed during the catalytic cracking of naphthalene (used as a tar model component). To the best of the authors’ knowledge, limited studies have simultaneously investigated the structural evolution and morphology of coke deposits on Fe-based catalysts, by comparing its formation in the presence or absence of steam. The novelty of this work lies in the combined use of a multitechnique approach together with kinetic deactivation analysis to directly relate coke nature and morphology to catalyst performance loss. Attention was given to correlating the degree of graphitization, ranging from amorphous to more ordered graphitic domains, with the extent of catalytic deactivation. These findings provide new insights into coke-induced deactivation mechanisms and support the design of more robust catalytic systems for tar reforming in waste gasification.

## 2 Materials and Methods

### 2.1 Catalyst Preparation

A schematic representation of the catalysts and their preparation methods is provided in Figure 1. Both catalysts were supported onto alumina support, which helps prevent the loss of active metals by strong interactions with it.^2^ Moreover, because of its high mechanical strength, chemical and physical stability, resistance to sintering, and high melting point, alumina is among the most commonly used catalytic supports for tar reforming reactions.^8,13,14^ The γ-Al_2_O_3_ used in this study is commercially available as 1 mm-diameter spheres, produced by Sasol Germany GmbH,^15^ and is suitable for these applications due to its high thermal stability.

**Figure 1:**
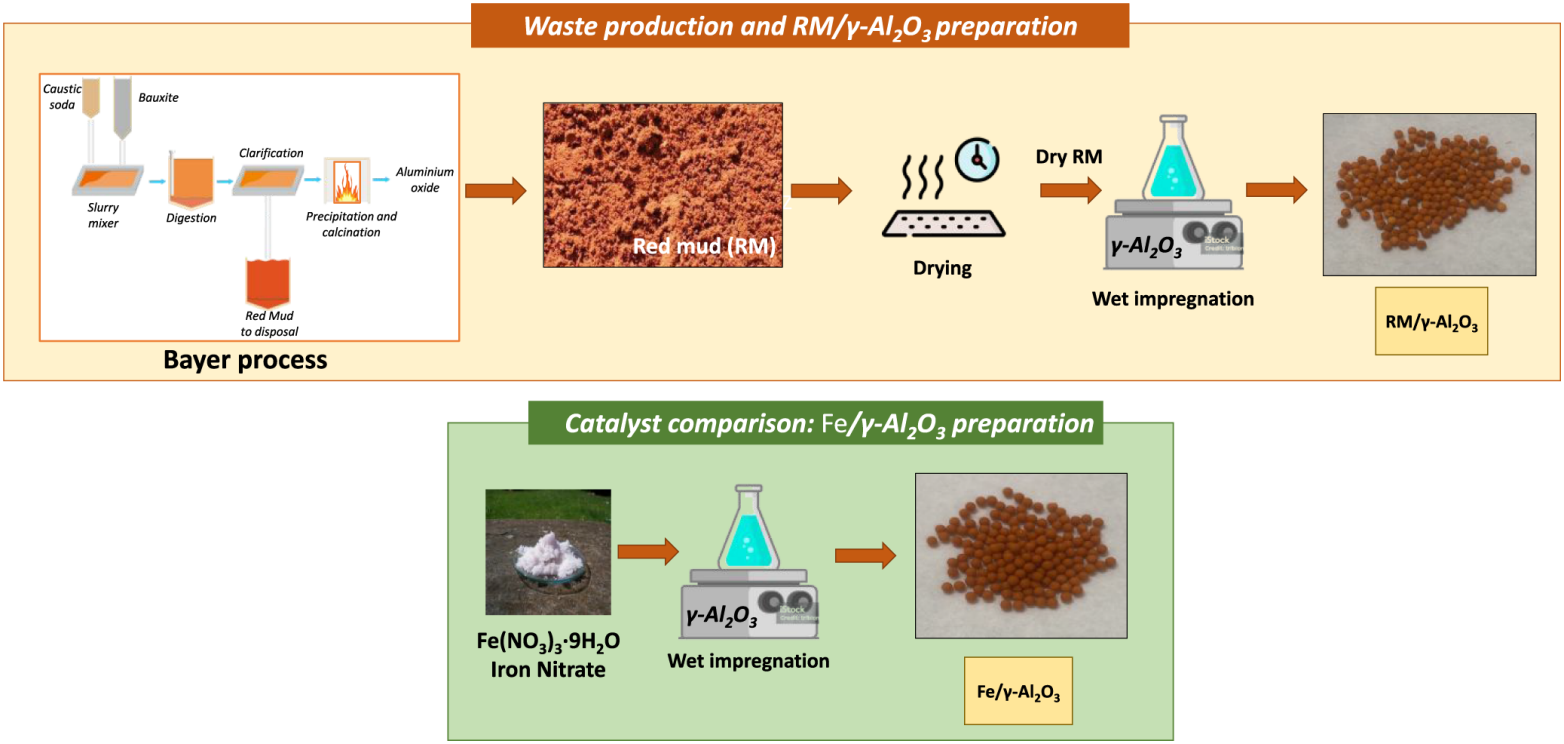
Sources and preparation procedure of the tested catalysts: top, RM/γ-Al_2_O_3_ (red mud supported by γ-alumina); bottom, Fe/γ-Al_2_O_3_ (iron supported by γ-alumina). Adapted with permission from Parrillo et al.^2^ Copyright 2024. Elsevier.

The other tested catalyst utilizes Red Mud (RM), an abundant waste produced from the Bayer process to extract aluminum from bauxite. The RM/γ-Al_2_O_3_ catalyst was prepared by wet impregnation, involving the dispersion of 5% red mud powder (50–100 μm) onto γ-Al_2_O_3_, followed by drying at 120 °C for 12 h and calcination at 800 °C in air. This temperature is below the γ-Al_2_O_3_ phase transformation range of this support (1000–1200 °C), and therefore the crystalline structure of the support is expected to be preserved. The catalytic performance of this waste-derived material was compared to that of a commercial iron-based catalyst supported on γ-alumina (Fe/γ-Al_2_O_3_), prepared by the same wet impregnation method. Fe/γ-Al_2_O_3_ was synthesized with an iron content of 3%_wb_ by dissolving the corresponding amount of iron nitrate nonahydrate Fe­(NO_3_)_3_·9H_2_O (98+%, Sigma-Aldrich) in an aqueous solution, followed by the addition of a γ-Al_2_O_3_ support. The mixture was then dried at 120 °C for 12 h and calcined at 800 °C in air. Although Fe in red mud is primarily present as Fe_2_O_3_, which is only weakly soluble in aqueous media, the wet impregnation route was intentionally selected because the objective was not to maximize the Fe loading, but obtaining a catalyst with a preparation procedure similar to that used for the Fe/γ-Al_2_O_3_ reference material, so making meaningful the comparison of catalytic performances.

### 2.2 Characterization of the Fresh Catalysts

The physical properties and inorganic compositions of the support and fresh catalysts are reported in Table 1.

**Table 1:** Physical Properties and Inorganic Composition of the Support, As-Received Red Mud and Fresh Catalysts^a^

Physical properties				
	γ-Al_2_O_3_ ^b^	RM	RM/γ-Al_2_O_3_	Fe/γ-Al_2_O_3_ ^c^
Catalysts diameter	1 mm			
Specific Surface Area (m^2^/g)	150–170	94.6	156	147
Pore Volume (cm^3^/g)	0.45	0.20	0.45	0.47
ICP MS analysis (mg/kg)^c^				
Li		6.0	0.3	-
Na		42160	1294	-
K		680	550.9	-
Al		1960	32490	
Zn		5.7	12.2	
Ti		21630	507	-
V		377.3	28	-
Cr		394.8	13.0	-
Mn	15	283.0	11.6	-
Fe	217	124300	3600	30000
Co		24.1	0.1	-
Cu	118	8.4	-	-

a Adapted with permission from Parrillo et al.,(2) Copyright 2024. Elsevier.

b Supplied by Sasol Germany GmbH.^15^

c Data from Parrillo et al.^7^

All the catalysts show specific surface areas of approximately 150–170 m^2^/g and, a pore volume exceeding 0.45 cm^3^ /g, which are like that of pure γ-Al_2_O_3_, highlighting the positive contribution of this support. RM/γ-Al_2_O_3_ has an iron concentration lower than that of Fe/γ-Al_2_O_3_, due to the limited solubility of iron species in the parent red mud matrix, as mentioned above. All elements, including Alkali and Alkaline Earth Metals (AEEM), are well distributed on the surface, as indirectly confirmed by the value of specific surface area that is rather close to that of pure γ-Al_2_O_3_.

### 2.3 Experimental Apparatus and Reaction Conditions

The tests were conducted in a bench-scale apparatus, shown in Figure S1 of the Supporting Information, and described in detail elsewhere^16^ together with the operating procedure. Briefly, the apparatus consisted of a tubular quartz reactor (14 mm i.d., 600 mm height) containing 2.6 g of catalyst, corresponding to a residence time of 0.11 s, which represents a good compromise between achieving high catalyst performance and avoiding issues related to operating limitations, such as excessive pressure drop.^16^ Naphthalene was used as a tar model compound and was vaporized in two bubblers kept at 65 °C, providing a concentration of 22.5 mg/L_N_. The tar model was carried into the reactor by a fixed flow rate of N_2_ (≈0.5 L_N_/min) and mixed with 7.5 vol % steam, supplied by a peristaltic pump and vaporized at 150 °C, before entering the reactor at 800 °C. All lines were heated to avoid condensation. The outlet gas was analyzed online by GC-TCD for permanent gases (H_2_, CO, CH_4_, N_2_, CO_2_, and light hydrocarbons) and periodically by GC–MS for residual naphthalene. Each test lasted 6 h. The spent catalysts were sampled after being cooled in an inert nitrogen-only environment and were weighed to quantify coke deposition. They were also analyzed by means of Raman spectroscopy, FT-IR, and SEM-EDX to assess the coke features.

The selected test conditions were representative of those measured at the outlet of a biomass/waste fluidized-bed gasifier.^17^ Tests were performed both with and without steam to evaluate the effect of the oxidant on the amount and nature of the coke formed.

### 2.4 Characterization Techniques

The Fe/γ-Al_2_O_3_ and RM/γ-Al_2_O_3_ catalysts were characterized to evaluate the coke formed during the process and its effect on the overall catalytic activity. FT-IR spectroscopy provided valuable insights into the presence and evolution of surface functional groups, especially oxygenated species such as carboxyl, lactones, hydroxyls, and carbonyls.^9^ These groups play a significant role in the interaction between the catalyst surface and the reactants or intermediate species and allow to identify possible chemical changes on the catalyst’s surface before and after the reaction. Before the FT-IR analysis, the catalysts were ground into a fine powder and homogenized with previously dried potassium bromide (KBr) to prepare 0.40 g disks containing 0.5% of the catalyst. This preparation ensured a homogeneous dispersion of the active phase in the IR-transparent matrix, improving spectral resolution and reducing scattering effects.^18^ The carbonaceous deposit was also analyzed by Raman spectroscopy, with particular attention to the identification of disordered carbon (D-band, ∼1350 cm^–1^) and graphitic structures (G-band, ∼1580 cm^–1^). Through the intensity ratio (*I*_D_/*I*_G_), indeed, it is possible to evaluate the degree of disorder and the structural evolution of coke, offering insights into the extent of catalyst deactivation due to carbon accumulation. These data are particularly valuable for correlating how the catalyst composition, the active phase, and the operating conditions affect the evolution of coke from amorphous to graphitic forms. In parallel, SEM-EDX analyses were carried out with the aim of cross-validating previous measurements and ensuring consistent interpretation of surface morphology and elemental composition. SEM images, coupled with EDX analyses, enabled the visualization of shape, surface roughness, and the presence of carbonaceous deposit, allowing direct evidence of morphological changes.^19^ EDX mapping, indeed, allowed for the semiquantitative determination of the elemental distribution, including the localization of active metals (mainly Fe), coke deposits or other byproducts. By correlating SEM-EDX data with FT-IR and Raman results, it is possible to assess changes in surface chemistry, carbon structure, and morphology, facilitating a better understanding of the mechanisms leading to deactivation and coke formation.

### 2.5 Deactivation Kinetics

The deactivation of both catalysts due to coke deposition was modeled assuming first-order decay in catalyst activity with respect to time-on-stream, following a lumped empirical approach as proposed by Levenspiel.^21^ Although it is known to depend on the composition of reactants, intermediates, and products, such effects are not explicitly included in the kinetic expression, but are implicitly accounted for in the apparent deactivation rate constant *kd*. Therefore, time-on-stream is commonly used as a practical variable to describe the progressive decay of catalyst activity in time-dependent deactivation models:^20^

1 $$a\left(t\right)=e^{-k_{d}t}$$

where *kd* [min^–1^] is the deactivation rate constant, and t [min] is the time on stream. In this model, the catalyst activity a­(t) decays exponentially according to Levenspiel^21^ and Jadon et al.^22^ By applying eq 1 to the mass balance equation of a packed bed reactor (PBR), the following expression is obtained:^21^

2 $$\ln\left(\ln\frac{C_{C10H8}^{0}}{C_{C10H8}}\right)=\ln\left(\tau^{\prime }k^{\prime }\right)-k_{d}t$$

where C^0^_C10H8_ is the inlet naphthalene concentration [mg/L_N_], C_C10H8_ is the outlet naphthalene concentration at different sampling time [mg/L_N_], τ’ is the “*weight time*” [g·min/L_N_], and k’ [L_N_/g·min] is the apparent reaction rate coefficient of naphthalene conversion per unit mass of catalyst, as discussed in the Supporting Information. Plotting the experimental data according to the eq 2 allows the determination of the deactivation rate constant *k*_d_, which corresponds to the slope of the resulting straight line. The experiments were conducted under kinetic control, and both external and internal mass transfer limitations were previously evaluated and found negligible, ensuring that the observed rates are not influenced by diffusional phenomena.^16^

## 3 Results and Discussion

### 3.1 Evolution of Hydrogen Concentration and Naphthalene Conversion

The results of hydrogen evolution and naphthalene conversion for Fe/γ-Al_2_O_3_ and RM/γ-Al_2_O_3_ catalysts are directly linked to the investigation of coke formation; hence, they are summarized in Figure 2. They are part of those detailed by Parrillo et al.^2^ and are included here for the sake of clarity, as they facilitate the discussion of coke deposition phenomena. The kinetic study of each catalyst’s deactivation is also introduced in this section to provide further insights into the extent and evolution of catalytic activity loss during the tests and their relationships with the observed coke deposition.

**Figure 2:**
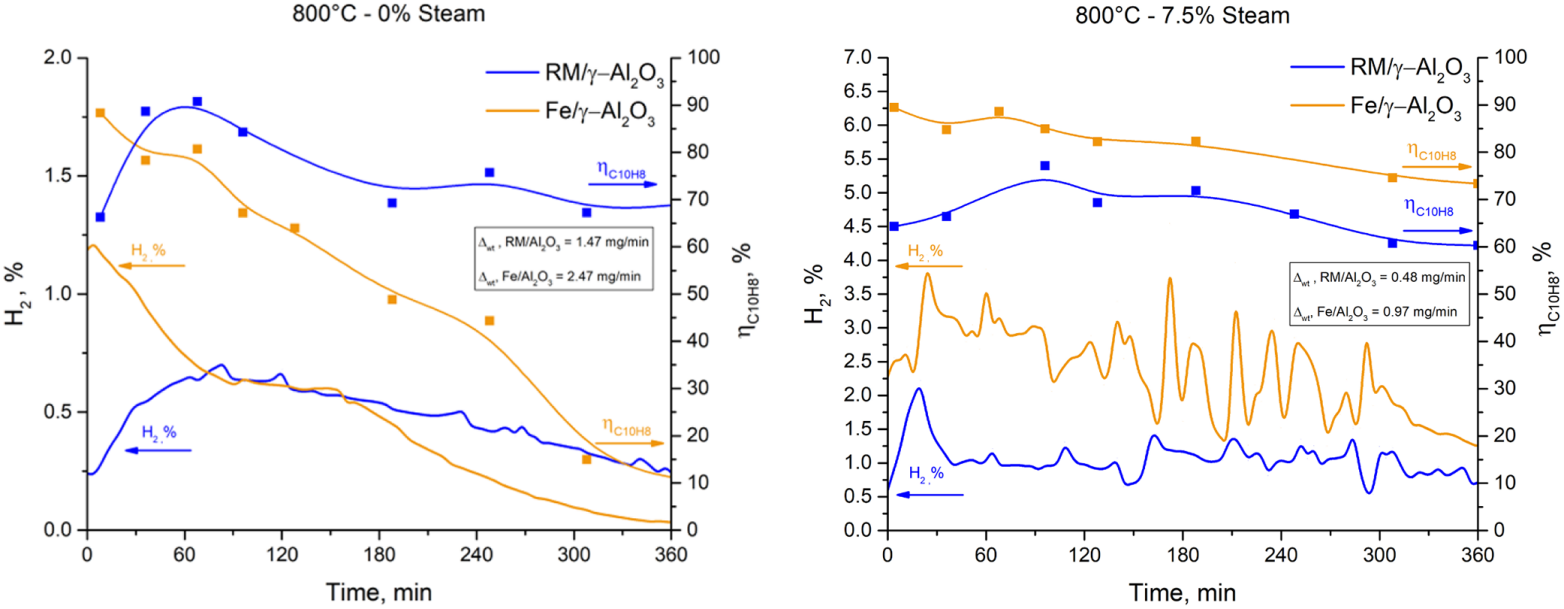
Time evolution of H_2_ (%_vol_) and naphthalene conversion at 800 °C, without and with 7.5% of steam, keeping the naphthalene concentration fixed at 22.5 mg/L_N_. Adapted with permission from Parrillo et al.,.^2^ Copyright 2024. Elsevier.

Fe/γ-Al_2_O_3_ exhibited the highest hydrogen production under all the tested conditions, due to its higher Fe content and the presence of metallic Fe already in the reduced state, which ensures high activity for C–C and C–H bond cleavage. The metallic Fe phase, indeed, provides electron-rich active sites that enhance its reactivity, while the absence of surface oxygen species minimizes steric hindrance.^23^ However, in the tests without steam, a marked deactivation was observed after 180 min, due to coke deposition. One possible explanation could be that the preparation method of this catalyst, using soluble iron nitrate, allowed Fe to penetrate deeper into the pores, making these sites rapidly inaccessible as coke accumulated. RM/γ-Al_2_O_3_ maintained instead a more stable performance over time, even though with an initial lower absolute hydrogen yield, being partly related to H_2_ consumption to reduce iron oxides present on the catalyst surface.^2^ In the tests without steam, the deactivation rate constant of Fe/γ-Al_2_O_3_ was 0.0032 min^–1^, which confirms its stronger susceptibility to coke deactivation, being significantly higher than the constant 0.0022 min^–1^ evaluated for RM/γ-Al_2_O_3_. The regression plots used to extract these deactivation rate constants are reported in Figure S.2 of the Supporting Information. In the tests with steam, both hydrogen yield and naphthalene conversion of Fe/γ-Al_2_O_3_ were higher than those of RM/γ-Al_2_O_3_, consistently with its higher iron content. Under these conditions, however, the measured deactivation rate constants of the two catalysts became very similar (around 0.0018 min^–1^), indicating that steam mitigated coke accumulation by promoting its partial gasification. In any case, a deactivation process is always present: residual coke was detected on both catalysts after testing. This interpretation is further supported by the quantified coke formation rates reported in Parrillo et al.,^2^ which, in the absence of steam, were 2.4 and 1.8 mg/min for Fe/γ-Al_2_O_3_ and RM/γ-Al_2_O_3_, respectively, that decrease to 0.98 and 0.5 mg/min in the presence of steam.

### 3.2 Evolution of Catalyst Morphology and Coke Characterization

#### 3.2.1 Insights into the Nature and Reactivity of Coke Deposits

The chemical nature and reactivity of carbonaceous deposits formed on the catalysts under different operating conditions were investigated through vibrational spectroscopies, FT-IR and Raman. The combined use of these analyses investigates the chemical nature of coke deposits and their degree of structural order, providing complementary information on coke reactivity and its impact on catalyst deactivation. Figure 3 compares the FT-IR spectra of the fresh and spent Fe/γ-Al_2_O_3_ and RM/γ-Al_2_O_3_ with those of the γ-Al_2_O_3_ support. The latter displays the typical broad band at 3480 cm^–1^, associated with the O–H stretching vibrations of surface hydroxyl groups and strong signals in the low-frequency region at 830 cm^–1^ and 570 cm^–1^, associated with the Al–O stretching vibrations in the γ-Al_2_O_3_ lattice.^24^ The spectra of the fresh Fe/γ-Al_2_O_3_ and RM/γ-Al_2_O_3_ catalysts do not show any additional features compared to those of the pure support, due to the low concentration of other compounds relatively to the total catalyst mass. In contrast, the spent catalysts exhibit some differences in the bands between 1300–400 cm^–1^ and 3100–2100 cm^–1^ under both operating conditions, attributed to the formation of different carbonaceous deposits. A deeper analysis, with magnifications of the 1300–400 cm^–1^ and 3100–2700 cm^–1^ ranges, is reported in Figures 4 and 5, respectively.

**Figure 3:**
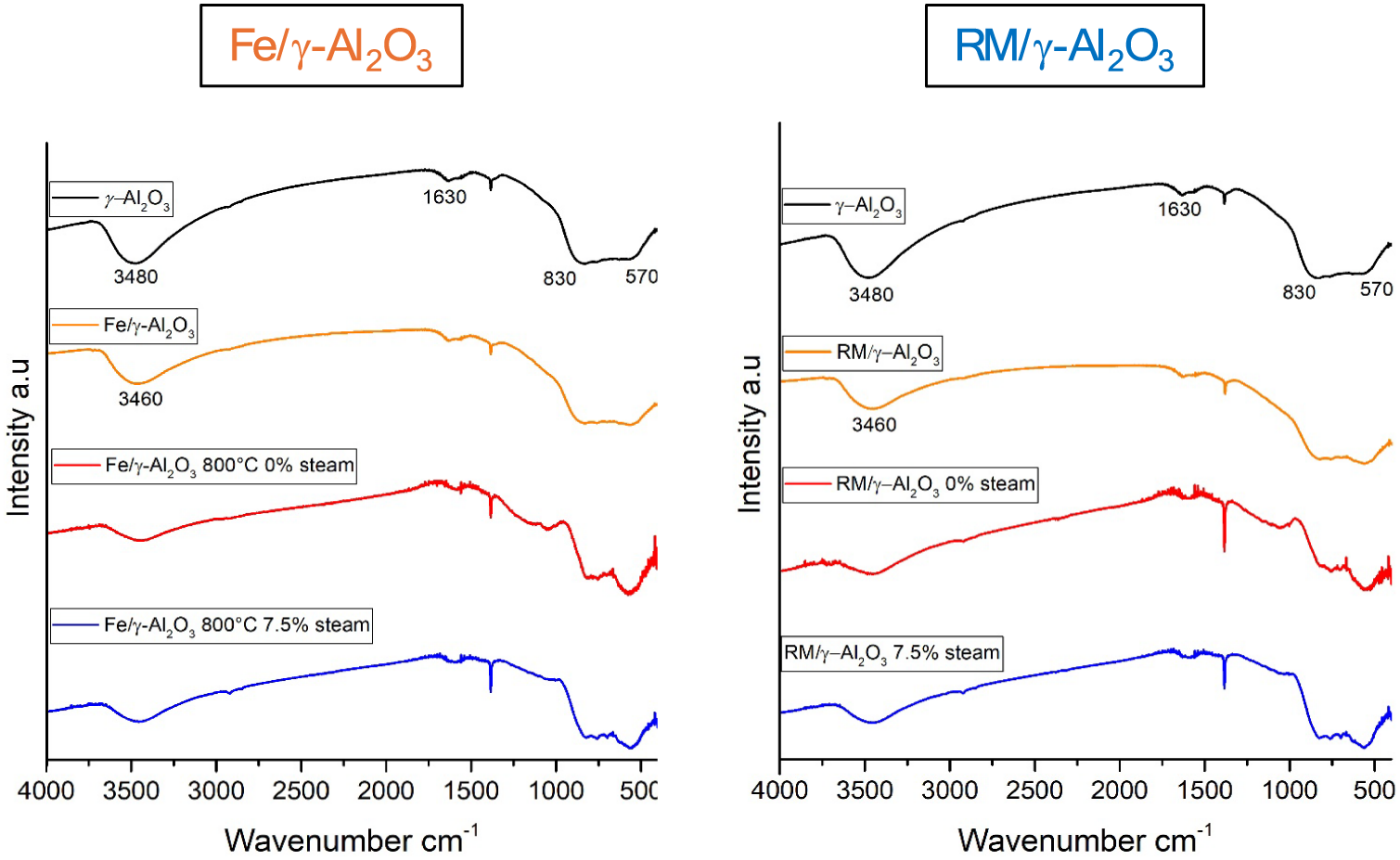
FT-IR of Fe/γ-Al_2_O_3_ and RM/γ-Al_2_O_3_ after tests without (red) and with (blue) steam, compared with those of the support (black) and the fresh material (yellow).

**Figure 4:**
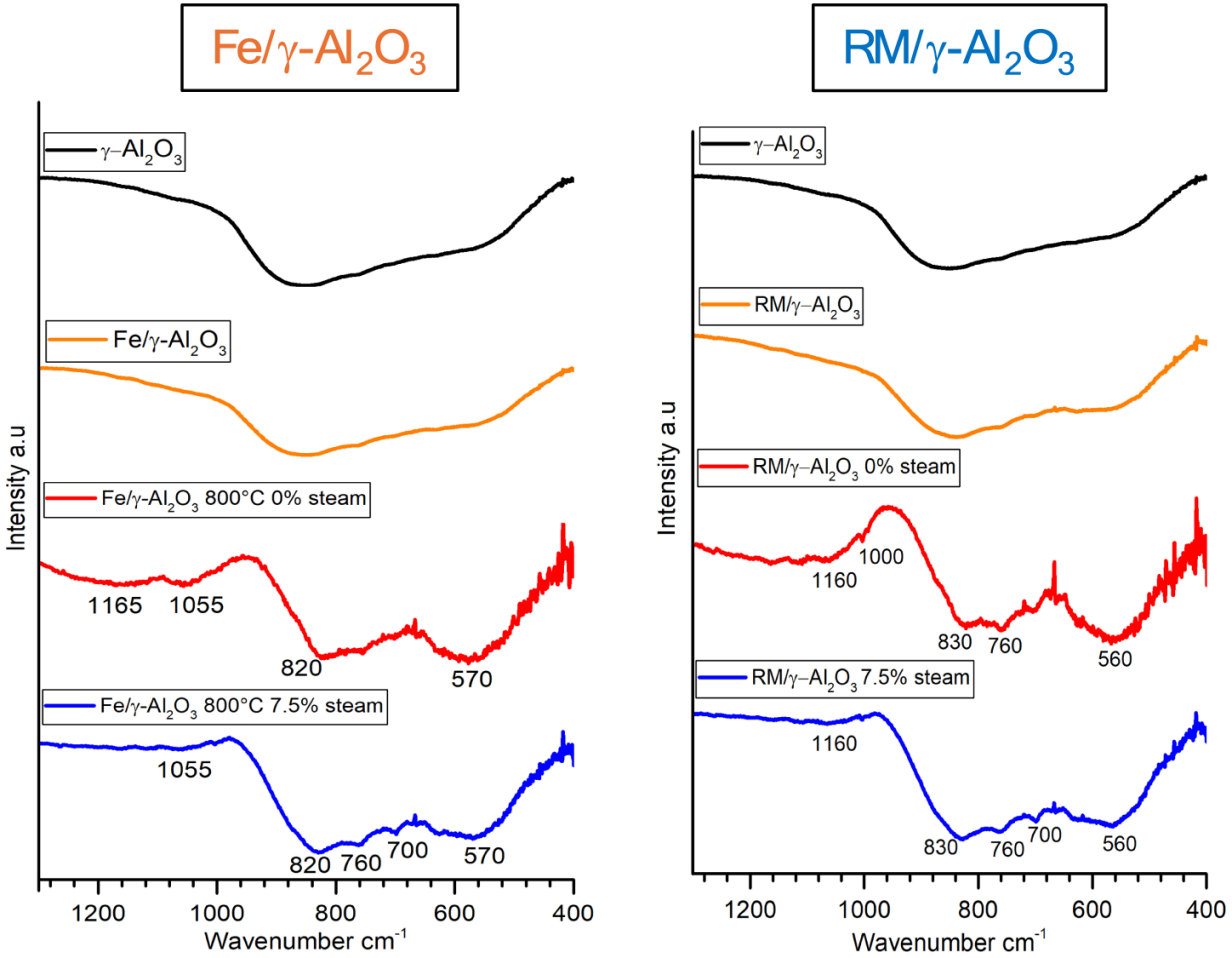
Magnification of the FT-IR spectra in 1300–400 cm^–1^ range, after tests without (*red*) and with (*blue*) steam, compared with those of the support (black) and the fresh material (*yellow*).

**Figure 5:**
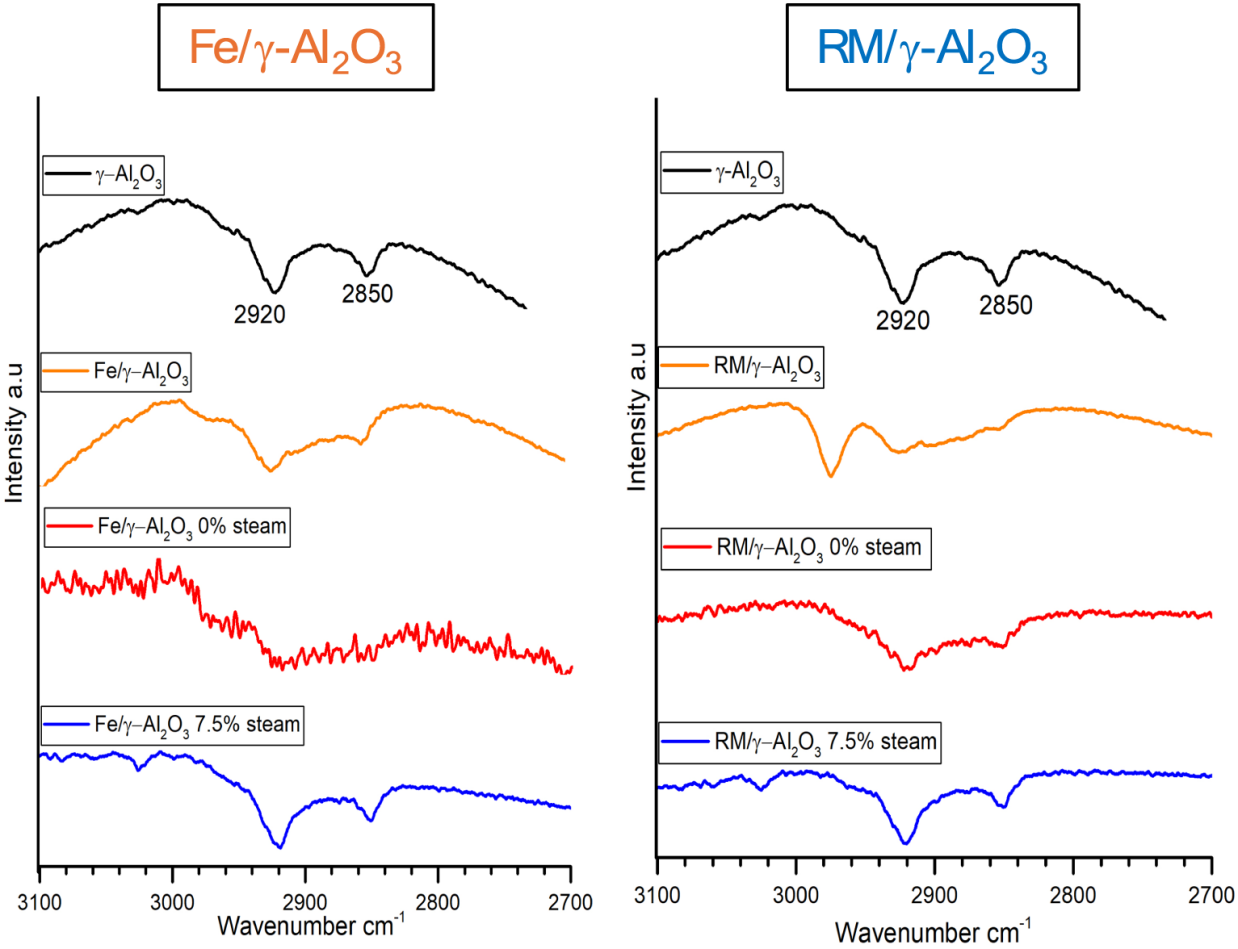
Magnification of the FT-IR spectra in 3100–2700 cm^–1^ range, after tests without (*red*) and with (*blue*) steam, compared with those of the support (black) and the fresh material (*yellow*).

The spectra of the support and fresh catalysts in the top part of Figure 4 do not show any significant peak. On the contrary, the spectra of both spent catalysts can be attributed to the in-plane vibrations of aromatic C–C and C–H bonds, which are characteristics of polyaromatic coke deposits.^25,26^ Figure 5 compares the spectra of the support, fresh, and spent catalysts in the 3100–2700 cm^–1^ range to assess the presence of aliphatic chains and aromatic rings due to the carbonaceous deposit.^27^ The support and the fresh catalysts show distinguishable bands at 2920 and 2850 cm^–1^, corresponding to the support signal, which are also visible in the catalysts tested in the presence of steam, indicating that a fraction of coke is effectively removed under these conditions. On the other hand, under tests without steam (red curves), the characteristic signals of the support are almost completely obscured, because coke deposits, including aliphatic and aromatic carbonaceous species, produce vibrational modes, which overlap the vibration of the support.

These observations suggest that two distinct coke fractions are formed. Steam can remove only the more reactive, aliphatic fraction of the deposited carbon, while the remaining fraction can be identified as aromatic and pyrolytic coke, produced through the cracking of heavier hydrocarbons, such as naphthalene.^28^ While differences are observed when varying the operating conditions, no significant differences are observed between the RM/γ-Al_2_O_3_ and Fe/γ-Al_2_O_3_ catalysts, likely because FT-IR primarily probes the aliphatic and aromatic functional groups of the coke, which are similar for both materials. Differences in the structural ordering of the coke are therefore largely masked.

The structural organization of coke deposits, beyond their chemical nature, plays a crucial role in determining their reactivity and impact on catalyst deactivation. The degree of order of the carbonaceous species was assessed through Raman spectroscopic indicators related to carbon structure. For the spent Fe/γ-Al_2_O_3_ catalyst tested without steam, the analysis reveals the coexistence of carbon structures with different degrees of order (Figure 6 top). Two distinct clusters can be identified based on their ID/IG ratios. The first cluster, characterized by an ID/IG ratio of 1.40 (Figure 6 top-left), corresponds to highly disordered and amorphous carbon, while the second cluster, with an ID/IG ratio of 0.84 (Figure 6 top-right), indicates a more ordered, graphitic-like structure. The presence of 2D bands in both clusters suggests poorly ordered multilayer carbon structures,^29^ which may be associated with potentially reversible forms of catalyst deactivation. When steam is fed, the structural characteristics of the remaining coke change markedly (Figure 6 bottom). The ID/IG ratios decrease to values slightly below 1, which is characteristic of more ordered carbonaceous structures. This can be attributed to the greater reactivity of a more amorphous coke, which leaves a more graphitic fraction, after the reaction with steam, as suggested by the absence of the 2D bands.^7^ These distinctions, further supported by FT-IR analysis, are relevant because, although both amorphous and graphitic carbon deposits contribute to catalyst deactivation, the enrichment of a more ordered, graphitic fraction under steam conditions may lead to a more persistent form of deactivation due to its lower reactivity.

**Figure 6:**
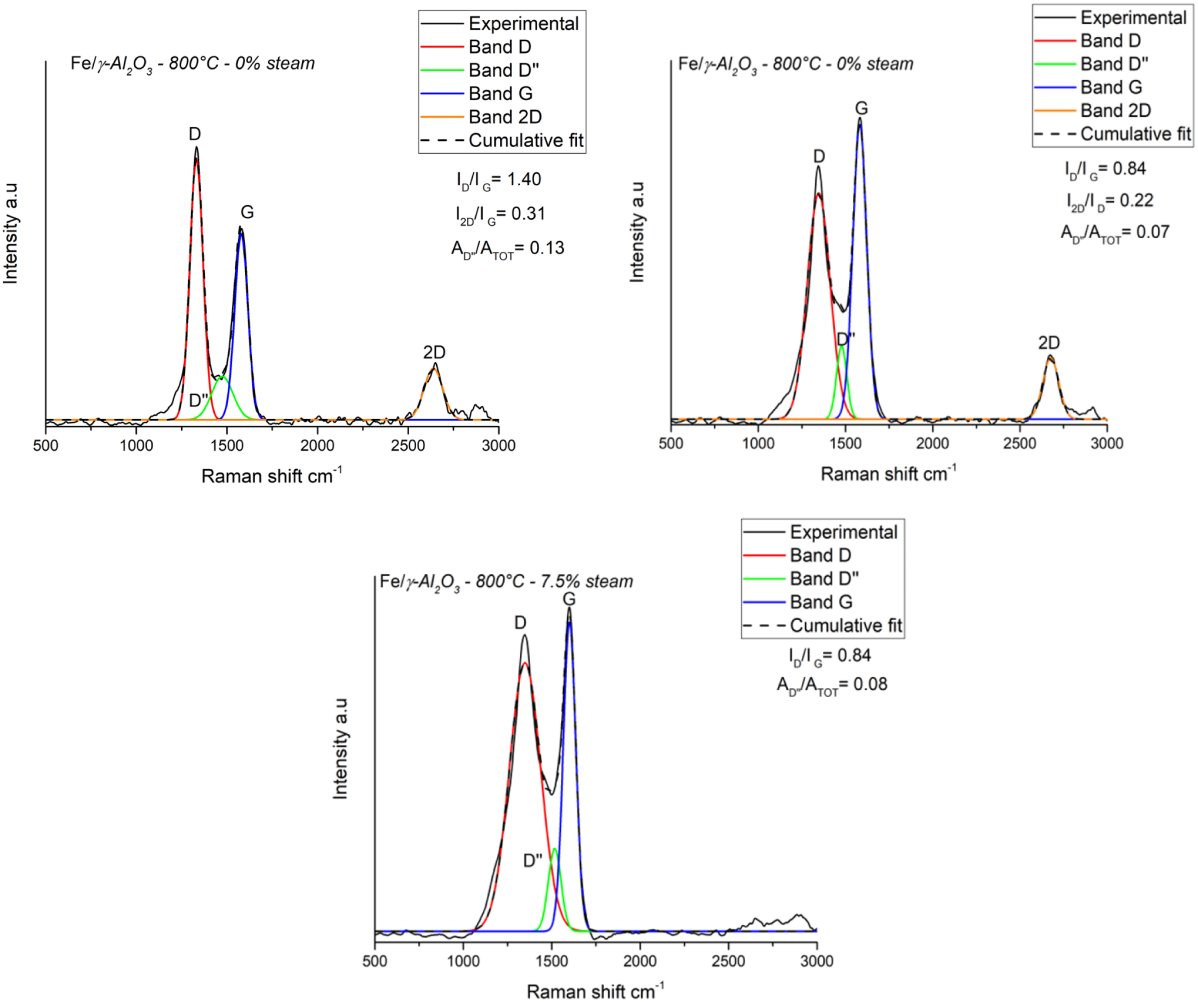
Raman spectroscopy of the Fe/γ-Al_2_O_3_ catalyst, without (top) and with (bottom) steam.

The Raman spectra for the RM/γ-Al_2_O_3_ are reported in Figure 7. As in the case of Fe/γ-Al_2_O_3_, the analysis of the samples without steam reveals the coexistence of carbonaceous deposits with different structural characteristics. A first cluster is characterized by a high I_D_/I_G_ ratio of 1.4, consistent with the predominance of highly disordered and amorphous carbon (Figure 7, top-left). In contrast, the second cluster exhibits a lower I_D_/I_G_ ratio of 0.96 and a reduced amorphous fraction (A_D″_/A_TOT_ = 0.05), highlighting the presence of more ordered carbon domains (Figure 7, top-right). The tests with steam show spectra with lower values of *I*_*D*_ */I*_*G*_ (0.87 and 0.96), combined with very low D″ contributions (A_D″_/A_TOT_ = 0.02 and 0.03, respectively). This can be attributed to steam that effectively limits the accumulation of amorphous coke by means of the Water–Gas reaction (H_2_O + C(s) ↔ CO + H_2_), leaving behind a carbon fraction with a higher degree of order (Figure 7, bottom). As already observed for the Fe/γ-Al_2_O_3_, the amorphous carbon phase, being more reactive, is preferentially consumed under steam conditions, while the graphitic phase remains. Moreover, the presence of the 2D band is negligible or absent, even without steam. This could be related to the presence of the AAEMs, which decrease the surface acidity and thereby help to reduce the formation of poorly ordered multilayer carbon structures.^2,30,31^

**Figure 7:**
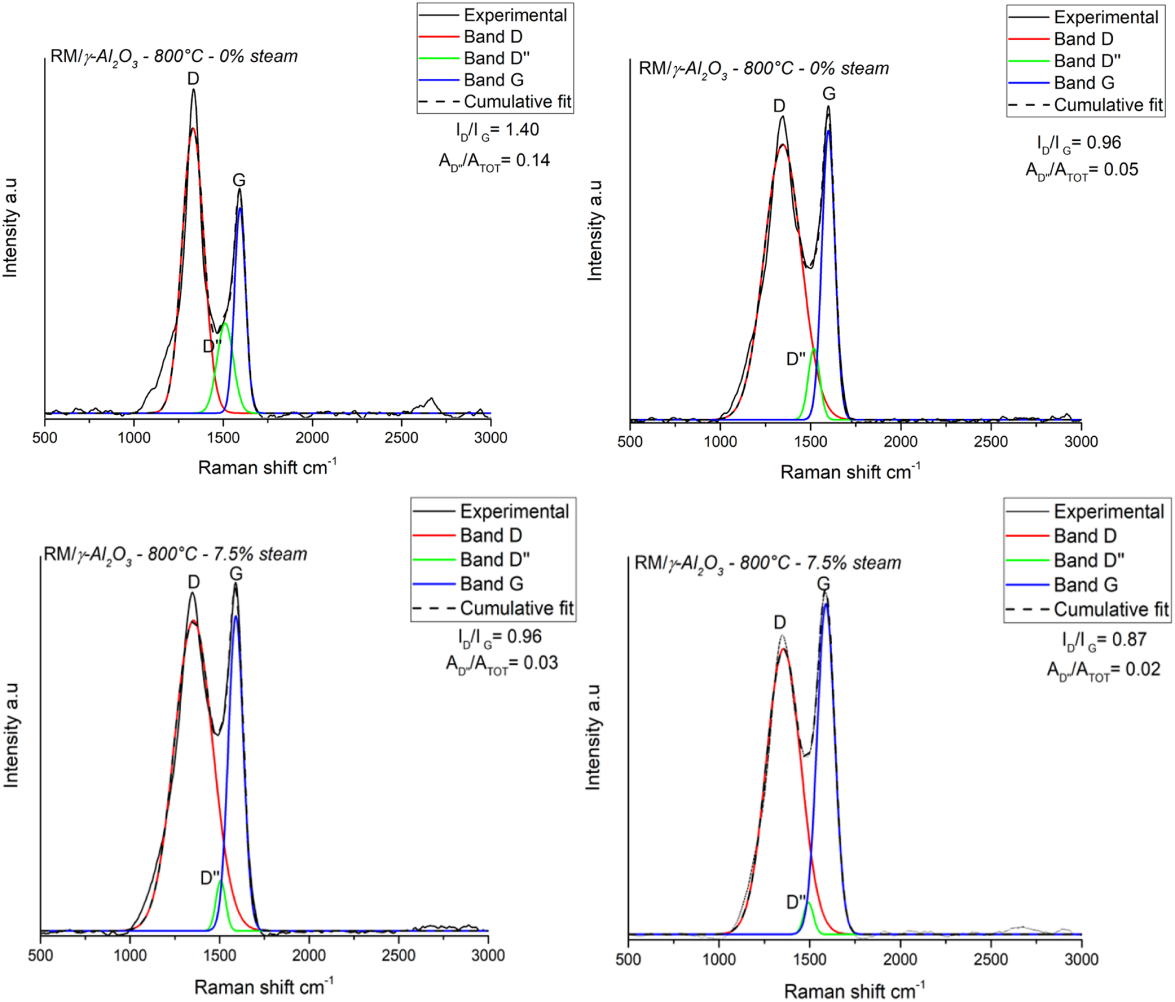
Raman spectroscopy of the RM/γ-Al_2_O_3_ catalyst, without (top) and with (bottom) steam.

#### 3.2.2 Distribution of Coke and Catalyst Morphology

The distribution of coke deposits and their interaction with the catalyst morphology and active phases were evaluated to provide qualitative insight into possible deactivation mechanisms (Figures 8 and 9). For the Fe/γ-Al_2_O_3_ catalyst (Figure 8), the fresh material exhibits two distinct surface regions: alumina-rich areas with lower iron concentration (zone 1) and localized iron-rich clusters dispersed on the support surface (zone 2). The EDX analysis of spent Fe/γ-Al_2_O_3_ shows a heterogeneous distribution of carbon: the areas with Fe clusters accumulate relatively less carbon than the surrounding alumina-rich regions. On a qualitative basis, this behavior may be correlated with Raman spectroscopy results, which indicate the coexistence of coke with different degrees of structural order on the catalyst surface. In this framework, Fe-rich clusters may preferentially interact with more disordered (amorphous) coke, which is generally considered more reactive. As a result, coke remaining on Fe-rich regions would tend to be relatively more graphitic, while the γ-Al_2_O_3_ support may retain both amorphous and graphitic coke, leading to a higher overall coke accumulation on alumina-rich areas.

**Figure 8:**
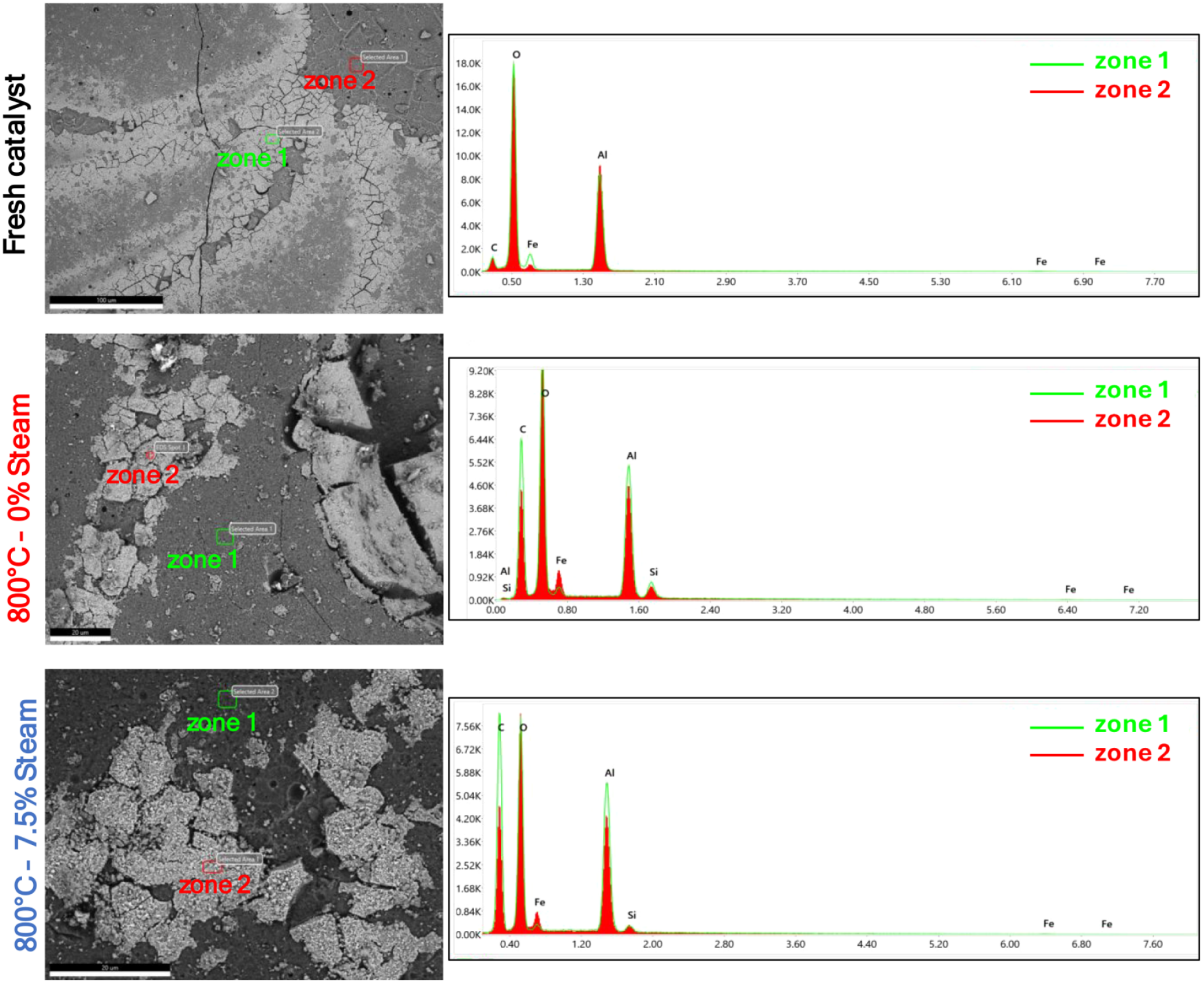
SEM-EDX analysis of Fe/γ-Al_2_O_3_, under different operating conditions.

**Figure 9:**
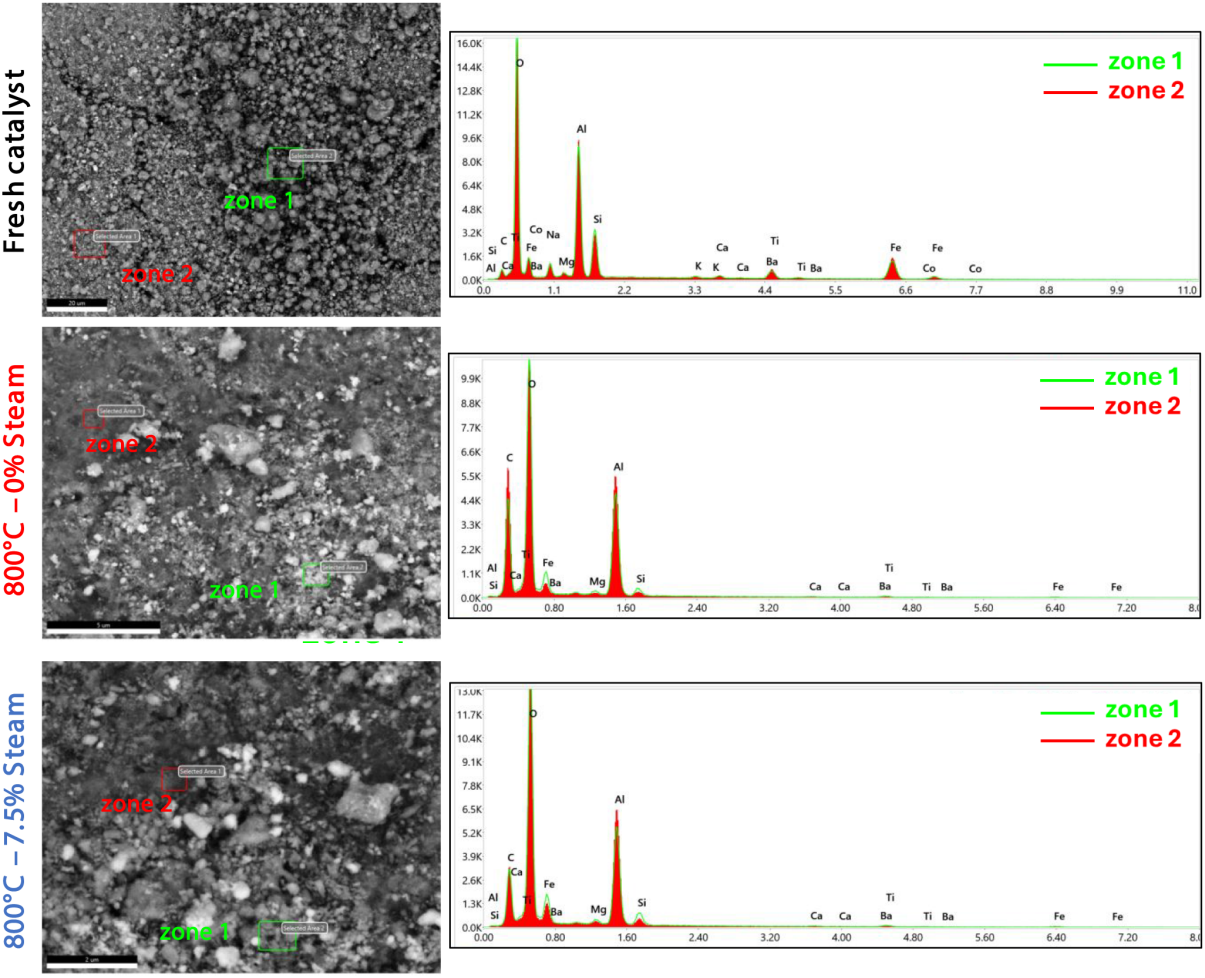
SEM-EDX analysis of RM/γ-Al_2_O_3_, at different operating conditions.

Without steam, the catalyst surface appears compact but with a distinct composition between the smooth zone 1 and the indented zone 2. EDX analysis reveals that zone 1 is dominated by Al and O of the support, whereas zone 2 is enriched in Fe of the active sites. In contrast, after treatment with 7.5% steam, the surface becomes apparently more granular and porous, with clear heterogeneity between different zones. Zone 1 still exhibits a composition dominated by Al and O, corresponding to the γ-Al_2_O_3_ support, whereas zone 2 is highly enriched in Fe. Although no clear decrease in carbon content is observed for the Fe/γ-Al_2_O_3_ catalyst when steam is introduced, reactions involving Fe redox cycle can facilitate carbon gasification, under these conditions.^32,33^ In parallel to the Water–Gas reaction, Fe may be oxidized by steam (3Fe + 4H_2_O ↔ Fe_3_O_4_ + 4H_2_) and oxidized iron can be reduced by solid carbon to regenerate metallic Fe and produce CO, so sustaining a redox cycle. The obtained CO—present in concentrations between 0.3% and 0.5% with steam and almost negligible without steam—may further participate in the Water–Gas Shift reaction or contribute to further reduce iron oxides, promoting partial recovery of active Fe sites. In contrast, the RM/γ-Al_2_O_3_ catalyst shows a markedly different morphology (Figure 9). The fresh catalyst does not exhibit clearly separated surface zones, and elements originating from red mud, such as Si, Ti, and trace AAEMs, are merged into the catalyst matrix. Unlike Fe/γ-Al_2_O_3_, the iron in RM/γ-Al_2_O_3_ appears more homogeneously distributed across the surface, suggesting a finer dispersion of the active phase. This feature may enhance the accessibility of active sites and reduce the formation of localized iron-rich clusters. Consequently, the SEM-EDX mapping of spent RM/γ-Al_2_O_3_ shows a more uniformly distributed carbon deposition across the surface, for both types of tests.

This characteristic, less pronounced in the Fe/γ-Al_2_O_3_ due to the presence of iron clusters, is typical of pyrolytic coke produced at high temperatures.^11,12^ Moreover, the persistence of red mud-derived elements after reaction suggests a rather good structural stability and their possible influence on catalytic performance.^30^ Overall, the SEM-EDX measurements seem indicate that RM/γ-Al_2_O_3_ exhibits a different deactivation profile compared to that of Fe/γ-Al_2_O_3_, with coke laying down more evenly across the surface, with an extent significantly limited under steam-assisted conditions.

### 3.3 Correlation of Multitechnique Data and Catalyst Performance Parameters

Multitechnique characterization and catalytic performance data jointly explain the relationships between catalyst structure, carbon deposition, and activity trends. Both catalysts demonstrate high efficiency and prolonged activity in the presence of steam, but coke buildup inevitably leads to deactivation during longer testing periods. The surface analyses reveal that different coke fractions, characterized by distinct chemical compositions and structural orders, play distinct roles in determining the catalytic behavior, and these roles correlate with the measured deactivation rate constants and hydrogen production trends. FT-IR spectra of spent catalysts revealed bands in the 1300–400 cm^–1^ and 3100–2100 cm^–1^ ranges, attributable to polyaromatic and aliphatic coke. The latter was absent in the presence of steam, indicating its potentially easy removal by steam, as evidenced by TGA and CHN analyses reported in Figure S.3 of the Supporting Information. In particular, the disappearance of aliphatic coke bands under steam conditions aligns with the lower deactivation rate constants (*k*_d_ = 0.0018 min^–1^) measured for both catalysts, suggesting that the removal of the more reactive carbon fraction mitigates the loss of activity. This interpretation is further supported by textural analysis, which revealed a decrease in specific surface area associated with pore blockage by carbon deposits. For Fe/γ-Al_2_O_3_, the surface area decreased by 32% and 12% without and with steam, respectively, whereas for RM/γ-Al_2_O_3_ the corresponding reductions were 25% and 22%. These results indicate, in agreement with previous literature studies,^34–36^ that coke deposition affects not only the chemistry of active sites but also the accessibility of the porous structure. Raman spectroscopy confirmed the FT-IR results, by highlighting the presence of both highly disordered and graphitic carbon on the two catalysts. Specifically, for catalysts tested without steam, higher ID/IG ratios indicate a predominance of amorphous carbon, which, together with a higher total coke deposition, correlates with faster deactivation. In contrast, for catalysts tested with steam, lower ID/IG ratios reflect more graphitic carbon, and the overall deposited coke is reduced due to partial gasification of the amorphous fraction, corresponding to slower activity loss. SEM-EDX analysis further supported these findings, showing localized Fe-rich clusters on Fe/γ-Al_2_O_3_ as preferential coke conversion sites, whereas RM/γ-Al_2_O_3_ exhibited a finer dispersion of iron and red mud-derived elements, contributing to a more uniform coke layer and better accessibility of active sites. Overall, although the semiquantitative nature of some characterization techniques limits exact numerical correlations, these results demonstrate that the structural characteristics of the catalysts—metal dispersion, support composition, and nature of carbon deposits—critically affect tar cracking efficiency and deactivation behavior. Fe/γ-Al_2_O_3_ is prone to localized graphitic coke accumulation, leading to severe deactivation, whereas RM/γ-Al_2_O_3_ exhibits more uniform coke deposition, enhanced structural stability, and a more moderate deactivation pathway, which is further modulated by steam and operating temperature.

## 4 Conclusions

A comprehensive investigation of Fe- and RM-based supported catalysts for tar cracking is provided, integrating detailed catalytic performance analyses with multitechnique characterization. The results indicate that the nature and distribution of carbon deposits, metal dispersion, and support composition critically influence both activity and deactivation pathways. Fe/γ-Al_2_O_3_ exhibited initially high activity, due to its higher Fe content and the presence of metallic Fe in the reduced state, which promotes C–C and C–H bond cleavage. However, it is susceptible to severe deactivation due to localized coke formation. In contrast, RM/γ-Al_2_O_3_ exhibits a more uniform coke deposition and enhanced structural stability. The negligible or absent 2D Raman band, even without steam, suggests that AAEMs may reduce the formation of the less-ordered coke layer, which is associated with a comparatively moderate deactivation pathway. The systematic correlation between multitechnique data (FT-IR, Raman, SEM-EDX) and catalytic performance parameters provides insights into the mechanisms of coke formation and its impact on activity. The adopted integrated approach made possible the identification of factors controlling catalyst stability, including the role of AAEM elements and the influence of steam in mitigating coke accumulation, which could also affect the iron oxidation state. These findings might have important implications for the design of more reliable syngas cleaning and tar conversion catalysts, even when these are derived from waste materials like red mud. Overall, the results underscore the importance of combining detailed structural characterization with catalytic testing to develop next-generation catalysts with improved performance and durability.

## Supplementary Material



## Notes

(i) A schematic overview and additional details of the experimental setup used for catalytic testing (S.1 – further details on the experimental apparatus); (ii) the methodology adopted for the determination of the deactivation constant (kd), including regression plots and discussion of the kinetic parameters (S.2 – determination of the deactivation constant kd); and (iii) complementary thermogravimetric (TGA) and CHNS analyses, providing further insight into coke deposition and carbon content on the investigated catalysts (S.3 – thermogravimetric and CHNS analysis) (PDF)

## Abbreviation

MSW: municipal solid waste
FT-IR: Fourier transform infrared spectroscopy
SEM-EDX: scanning electron microscopy combined with energy dispersive X-ray analysis
AAEM: alkali and alkaline earth metals
RM: red mud; GC-TCD
Gas: chromatographer coupled with thermal conductivity detector
GC-MS: gas chromatographer coupled with a mass spectrometer
