# Supplementary material for: Catalyst Deactivation in Syngas Tar Cracking: A Multianalysis Study of Coke Deposition on γ‑Al2O3‑Supported Iron Catalysts
Source: ACS Omega. 2026 Jun 16;11(25):37378–89. doi: 10.1021/acsomega.6c01836 (PMC13325162; doi:10.1021/acsomega.6c01836)
Supplement: Supplementary file 1 [file ao6c01836_si_001.pdf]

CATALYST DEACTIVATION IN SYNGAS TAR CRACKING: A MULTI-ANALYSIS STUDY OF COKE DEPOSITION ON γ-Al_2O_3-SUPPORTED IRON CATALYSTS

Francesco Parrillo[1*], Vincenzo Arconati[1], Carmine Boccia[1], Umberto Arena[1], Filomena Ardolino[1], Giovanna Ruoppolo[2], Ange Nzihou[3], María González Martínez[3]

[1] Department of Environmental, Biological, Pharmaceutical Sciences and Technologies – University of Campania "Luigi Vanvitelli", Via Vivaldi 43-81100, Caserta, Italy
[2] Institute of Sciences and Technologies for Sustainable Energy and Mobility, National Research Council-CNR. P.le Tecchio 1, 80125, Naples, Italy
[3] Université de Toulouse, IMT Mines Albi, RAPSODEE CNRS UMR 5302, Campus Jarlard, F.81013, Albi, Cedex 09, France

*Corresponding author: francesco.parrillo@unicampania.it

SUPPLEMENTARY INFORMATION

S.1 – Further details on the Experimental Apparatus

Figure S.1 provides a schematic overview of the laboratory-scale setup used in this work, to support the experimental discussion reported in the main text. The system includes a catalytic fixed-bed reactor with controlled nitrogen and steam supply, a condensation line for tar recovery, and dedicated analytical devices (GC–MS and GC–TCD) for the characterization of both condensable and permanent gas products. This schematic representation provides a general idea of the experimental apparatus. Further details are provided in Arconati et al., 2025.

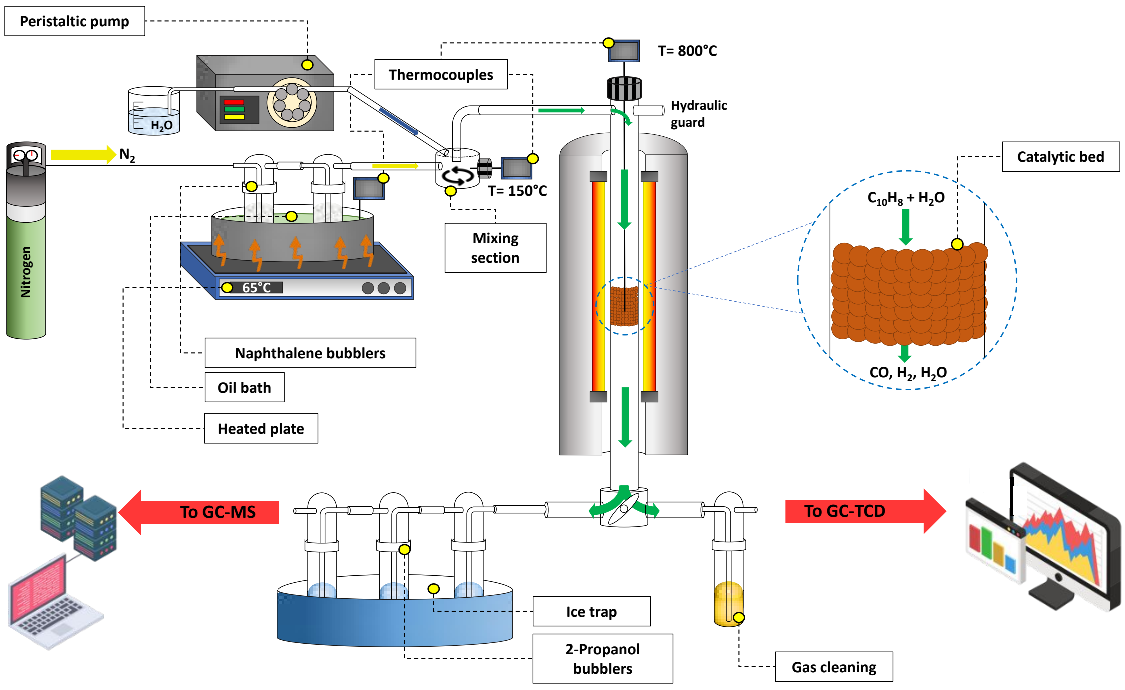

Figure S.1 Schematic of the experimental apparatus.

S.2 – Determination of the Deactivation Constant k_d

The deactivation constant k_d, was determined by fitting the experimental data to a linear model describing the decay of catalytic activity over time on stream. The slope of the resulting regression line corresponds to the value of k_d, while the intercept provides a measurement of the reaction rate coefficient k', as reported by Levenspiel, (1999). The plots and fitted lines used to extract the deactivation and the reaction rate coefficients for the different operating conditions and catalyst formulations are shown in Figure S.2. It is important to note that the k'values obtained from these plots correspond to the initial apparent activity of the fresh catalyst at zero time-on-stream. This method typically yields slightly higher values of k'compared to those calculated using the integral method, by applying the PBR performance equation (Arconati et al., 2025). In the integral method-PBR approach, k' values were calculated using the initial naphthalene conversion data, minimizing the impact of progressive catalyst deactivation.

Despite these differences, the overall trend remains consistent: slightly higher k' values are obtained under dry conditions (0.44 $L_N/(g \cdot min)$-both catalysts) compared to tests performed in the presence of steam (0.40–0.25 $L_N/(g \cdot min)$ for Fe/γ-Al_2O_3 and RM/γ-Al_2O_3, respectively). This observation aligns with a previous study and could be explained by the different iron oxidation state. Without steam, reduced iron, which is more active than oxidized iron, was present at higher concentration than that in the presence of steam (Arconati et al., 2025).

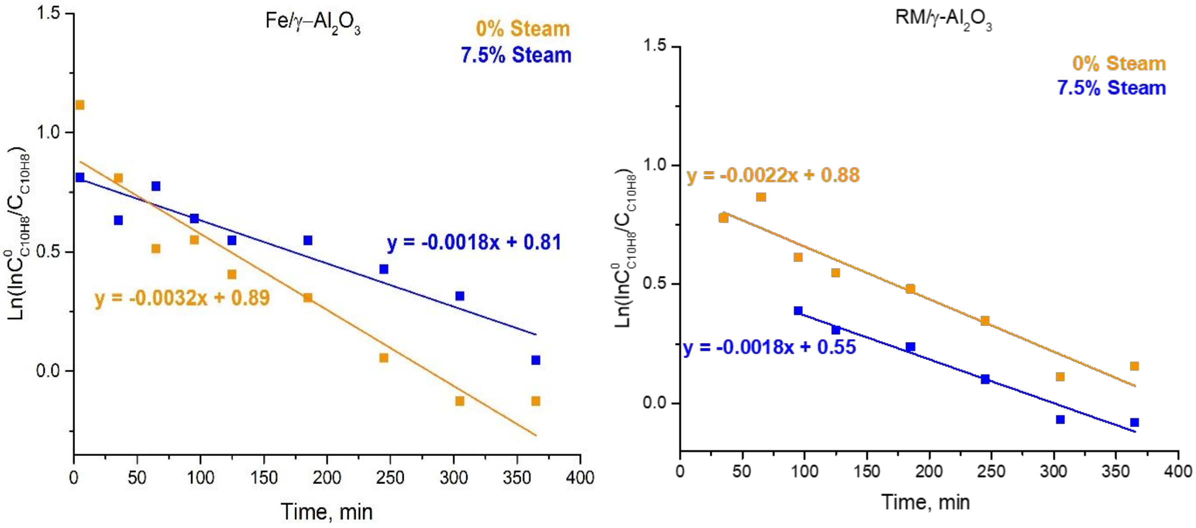

Figure S.2 Extrapolation of the deactivation constant k_d at 800°C: orange curve for the test without steam and blue curve for the test with steam

S.3 – Termogravimetric and CHNS analysis

Figure S.3 reports the curves obtained by TGA with air, together with data of total mass loss (Δwt), measured by TGA, and carbon content, measured by CHN analyses. The fresh catalysts show only an initial weight loss around 100°C, due to the evaporation of adsorbed water (RM/γ-Al_2O_3 about 5 %, Fe/γ-Al_2O_3 about 2.5 %). The TGA of RM/γ-Al_2O_3 and Fe/γ-Al_2O_3 show a comparable weight losses in the absence of steam: 28.4 % for RM/γ-Al_2O_3 (with a C content of 26.5 %) and 27.4 % for RM/γ-Al_2O_3 (with a C content of 22 %). These weight losses are mainly attributed to the presence of a coke layer. Fe/γ-Al_2O_3, in particular, shows two different temperature of mass losses (400°C, without steam, and 550°C, with steam), confirming different structures of coke, which could deposit on different parts of the surface, as obtained by multi-analysis reported in the main text.

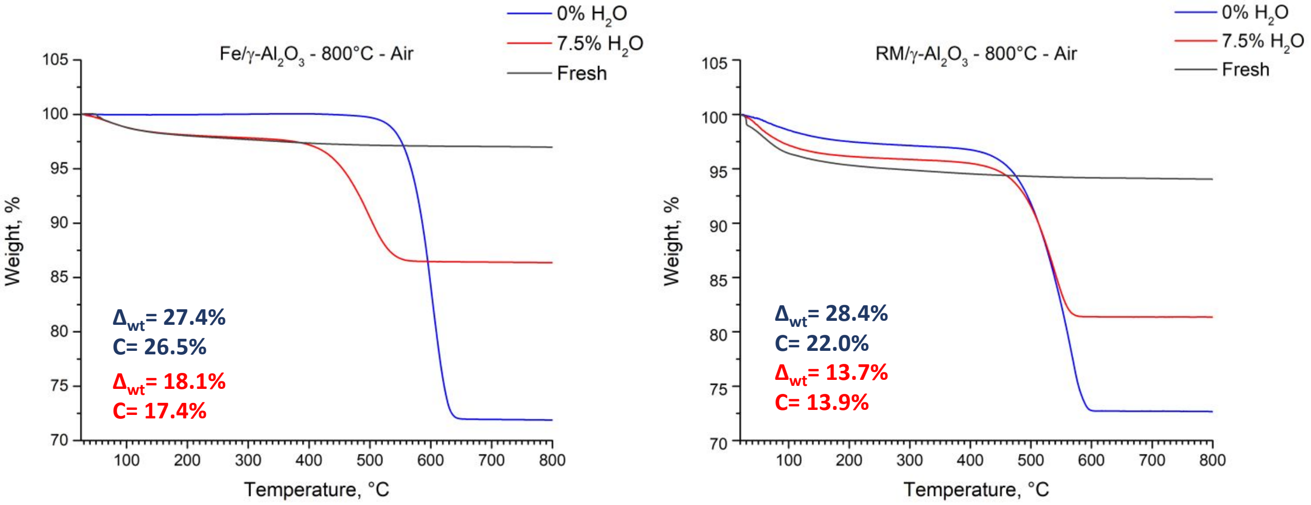

Figure S.3 Thermogravimetric analysis of the different catalysts tested at 800 ◦C, without and with 7.5 % of steam.

REFERENCES

Arconati, V., Boccia, C., Ardolino, F., Ruoppolo, G., Arena, U., Parrillo, F. 2025. Design of a tar catalytic cracker based on hot syngas clean-up tests. Chemical Engineering Research and Design. 271, 152-161. https://doi.org/10.1016/j.cherd.2025.03.022

Levenspiel, O., 1999. Chemical reaction engineering. 3rd edition. J. Wiley and Sons ISBN: 0-471-25424-X
